# Retrench or Bankrupt—Comparative Cost of the Transactions Each Year

**Published:** 1892-05

**Authors:** 


### EDITORIAL DEPARTMENT.

F. E. DANIEL, M. D., EDITOR AND PUBLISHER.

THIS JOURNAL, by unanimous vote of the members, was made the Official Organ of the Austin District Medical Society.

COLLABORATORS:

*Dr. R. M. Swearingen, Austin.*
*Prof. B. E. Hadra, M. D., Galveston.*
*Prof. Geo. Cupples, M. D., San Antonio.*
*Dr. T. C. Osborn, Cleburne, Texas.*
*Dr E. J. Doering, Chicago.*
*Dr. E. J. Beall, Fort Worth.*
*Dr Odo Betz, Germany.*
*Prof. W. B. Rogers, M. D., Memphis.*
*Dr. J. W. McLaughlin, Austin.*
*Dr. T. J. Tyner, Austin.*
*Prof. J. F. Y. Paine, M. D., Galveston.*
*Dr. R. H. L. Bibb, Mexico.*
*Dr. T. J. Bennett. Austin.*
*Dr. Bat Smith, Wharton.*
*Dr. E. Meierhof, New York.*
*L. H. Luce, M. D., West Tisbury, Mass.*

### RETRENCH OR BANKRUPT.

While the majority of the members of the State Association are subscribers to the JOURNAL, and are interested in the Association's finances, they constitute but a small part of the list; and we must apologize to our other readers for devoting so much space to Association matters.

By reference to the proceedings it will be seen that Dr. Q. C. Smith's resolution to repeal the ordinance setting aside $300 each year to pay the Publishing Committee, was tabled. This sum is in addition to the Secretary's salary of $200, and the Secretary, being chairman of the Publishing Committee and doing this work, receives the $300. It is nothing but right he should be paid this sum; the work is worth it; if the finances of the Association were in condition to justify it.

Dr. Smith, before offering his resolution, should have explained the condition of the finances; the necessity for retrenchment would then have been apparent; otherwise he might have known the resolution would be defeated.

Very few members know or care anything about the finances, so there is enough to pay expenses; but if they will look into the matter, they will see that expenses have greatly increased, while the membership has decreased, and consequently receipts this year will be short.

Below is given a plain statement of facts; and it must be apparent that the Association must retrench or be bankrupt.

The report of the Treasurer at Waco, April 1891, shows that of the total on hand and collected to that date the amount paid out (under the old Publishing Committee) all told, was $1414; and a balance was left on hand of $448.

To this balance add the receipts at Waco and subsequently, up to the Tyler meeting, ($1877), and we have a total of $2325.

Under the new order of things all of this vast sum was paid out (to-wit, $2190), except—$130,—and for what? The items of disbursement are the same in each year, except $175 paid the stenographer for work, taking the minutes and preparing copy for the Transactions.

The main increase in expense seems to have been in the increased cost of the Transactions, which, notwithstanding the fact that it is the smallest volume since '84, and fewer copies were issued by fifty than ever before (since 1883), it will be seen that it cost per volume considerably more than double that of '84, double that of '89-90, and about 80 per cent. more than that of 1890-1, all in same binding, except that of '84, of which half (350) were in paper.

The attendance at Tyler was unusually slim, and fewer new members joined than for many years. The Secretary reports sixty dropped from the roll, and hence the amount collected at Tyler was small;--the amount likely to be collected yet, will be unusually small, and with only the $130 left in the hands of the Treasurer out of nearly $2400, it is demonstrated that something must be done.

The bill for the Transactions, paid by the Treasurer, is $795. This includes postage on 530 copies, some $63. Deduct this, and add the $175 paid stenographer, and it will be seen the cost of the 600 books, exclusive of postage, is $908, against $557* in 1889-90, and $508 in 1890-91; and we see no improvement.

The work could have been gotten out in Austin for much less money, as will be seen by reference to the subjoined comparison. And we know that the Austin printer, who for several years past

---

*In the Treasurer's report for that year the amount paid for Transactions is put down at $667. This is an error, and includes $115 paid for printing for Legislative Committee.—Ed.

has secured the contract on competitive bid, asked to be permitted to bid, but was refused.

COST OF TRANSACTIONS—(Including binding, wrappers, etc.)

| | No. Vols. | No. Pages. | Total Cost exclusive of postage. | Per Vol. | Per Page. |
|---|---|---|---|---|---|
| Fort Worth—1884-85 ....... | 700* | 246 | $475 00 | $ .68 | $1 48 |
| Austin—1889-90 (cloth) ..... | 650 | 346 | 507 00 | .78 | 1 49 |
| Austin—1890-91 " ..... | 650 | 343 | 554 20 | .85 | 1 61 |
| Galveston—1891-92 (cloth) ... | 600 | 278 | 733 00 | 1 51 | 3 26 |
| Stenographer's bill ....... | | | 175 00 | | |

The Transactions of 1884 was gotten out in Fort Worth, while Secretary Burt, chairman of committee, resided at Austin, Drs. Daniel and Broiles, at Fort Worth, being on the committee. The total cost for 700 volumes, one-half in cloth, and containing 246 pages was $475, the lowest price for which a decent volume has ever been issued (68c. per volume), and yet a member of the Publishing Committee was charged by a member, since expelled, with appropriating $75, and the Association seriously entertained the charge and investigated it. Yet, at Tyler they pay without hesitation or inquiry considerably more than double the cost per volume for 600 volumes of only 32 pages more.

If it be objected that the Transactions could not have been printed elsewhere than Galveston, last year, the above is cited as precedent; and in this case the old committee, who for seven years have gotten out the Transactions at Austin, all reside in Austin.

The *real* cost of the Association's volume of Transactions is gotten at thus:

| | |
|---|---|
| Salary of Secretary ................................ | $ 200 00 |
| Salary of Secretary as Chm'n Pub. Com ............. | 300 00 |
| Salary of Stenographer ........................... | 175 00 |
| Printer's bill .................................... | 733 00 |
| Postage, 600 at 12c ............................... | 72 00 |
| | $1480 00 |

or $2.47 per volume.

*Thus the cost of the Transactions* for 1891-92 exceeds the

*Half in cloth.

entire expense of the Association for 1890-91 by $66; while the total expense of the Association ($2190) exceeds that of the previous year by $776 in round numbers.

Would it not be well to restore the old Publishing Committee?

